# Genetic Landscape of Peripheral T-Cell Lymphoma

**DOI:** 10.3390/life12030410

**Published:** 2022-03-11

**Authors:** Vivian Hathuc, Friederike Kreisel

**Affiliations:** Department of Laboratory Medicine and Pathology, Saint Louis University, St. Louis, MO 63104, USA; vivian.hathuc@health.slu.edu

**Keywords:** T-Cell lymphoma, T-Cell receptor signaling, epigenetic regulators

## Abstract

Peripheral T-Cell lymphoma (PTCL) comprises a heterogenous group of uncommon lymphomas derived from mature, post-thymic or “peripheral” T- and natural killer cells. The World Health Organization (WHO) emphasizes a multiparameter approach in the diagnosis and subclassification of these neoplasms, integrating clinical, morphologic, immunophenotypic, and genetic features into the final diagnosis. Clinical presentation is particularly important due to histologic, immunophenotypic and genetic variations within established subtypes, and no convenient immunophenotypic marker of monoclonality exists. In recent years, widespread use of gene expression profiling and next-generation sequencing (NGS) techniques have contributed to an improved understanding of the pathobiology in PTCLs, and these have been incorporated into the 2016 revised WHO classification of mature T- and NK-cell neoplasms which now encompasses nearly 30 distinct entities. This review discusses the genetic landscape of PTCL and its role in subclassification, prognosis, and potential targeted therapy. In addition to discussing T-Cell lymphoma subtypes with relatively well-defined or relevant genetic aberrancies, special attention is given to genetic advances in T-Cell lymphomas of T follicular helper cell (TFH) origin, highlighting genetic overlaps between angioimmunoblastic T-Cell lymphoma (AITL), follicular T-Cell lymphoma, and nodal peripheral T-Cell lymphoma with a TFH phenotype. Furthermore, genetic drivers will be discussed for ALK-negative anaplastic large cell lymphomas and their role in differentiating these from CD30+ peripheral T-Cell lymphoma, not otherwise specified (NOS) and primary cutaneous anaplastic large cell lymphoma. Lastly, a closer look is given to genetic pathways in peripheral T-Cell lymphoma, NOS, which may guide in teasing out more specific entities in a group of T-Cell lymphomas that represents the most common subcategory and is sometimes referred to as a “wastebasket” category.

## 1. Introduction

The pathogenesis of peripheral T-Cell lymphoma is a complex process that largely involves the dysregulation of signaling pathways crucial for normal T-Cell development, differentiation, and virus-mediated oncogenesis. Alterations in the non-neoplastic microenvironment promoting lymphoma growth are not discussed in this review.

Normal T-Cell development starts in the bone marrow from a multipotent hematopoietic stem cell that gives rise to precursor T-lymphoblasts. These precursor blasts enter the thymic cortex as CD4/CD8 double negative early thymic progenitors, where they undergo the first step of thymic selection by developing a functional T-Cell receptor (TCR). Depending on the appropriateness of interactions with self-antigen-expressing thymic epithelial cells, these TCR-expressing and CD4/CD8 double positive thymocytes will then undergo negative or positive selection. Thymocytes with defective TCRs are removed by negative selection through apoptosis. Positively selected thymocytes migrate to the medulla to further differentiate into mature CD4 single positive or CD8 single positive T-lymphocytes. After thymic maturation, naive CD4 positive, and CD8 positive T-lymphocytes then circulate in peripheral blood and the lymphatic system to further differentiate into specialized CD4 and CD8 T-Cell subsets [1]. These express the alpha/beta T-Cell receptor and have undergone somatic V(D)J segment recombination to display the diversity and specificity to recognize a vast array of foreign antigens [2]. CD4 T-Cell subsets include regulatory and various helper T subsets (i.e., Th1, Th2, and T follicular helper subsets), and commitment to these subsets is dependent on the expression of certain transcription factors, known as master regulators [3]. FOXP3 expression is associated with differentiation into T regulatory cells, GATA3 expression is with differentiation into the Th2 subset, TBX21 (Tbet) expression is with differentiation into the Th1 subset, and BCL6 expression is with differentiation into the T follicular helper subset [3]. The expression of these transcription factors is regulated by specific cytokines, and cytokine stimulation in T-Cells is largely controlled by the Janus kinase/signal transducer and activator of the transcription protein (JAK/STAT) signaling pathway [4]. For example, GATA3 expression is induced by IL-4 regulated by STAT6, while TBX21 expression is induced by interferon Y (IFN Y) and IL-12 through STAT1 and STAT4, respectively. Interleukins (IL)-6 and -21 are regulated by STAT1 and STAT3, respectively, to promote BCL6 expression. The differentiation into specific T-Cell subtypes after antigen exposure ensures that the immune system is fine-tuned to fight infections and protect against autoimmunity. This requires a highly coordinated interaction between TCR, co-stimulatory, and cytokine signaling pathways. TCR/CD3 activation is triggered by antigen presentation through major histocompatibility complex (MHC) molecules and supported by co-stimulatory molecules, such as CD28, and tumor necrosis factor receptors (TNFRs), such as CD30, CD40, and OX40 [3]. Engagement of these co-stimulatory molecules leads to activation and transcription of signaling pathways important in T-Cell survival, maturation, and clonal expansion of normal T-Cells. The three main pathways activated through the TCR that control transcription are mitogen-activated protein kinase (MAPK), the nuclear factor kappa-light-chain-enhancer of activated B-cells (NF-kB) pathway, and the calcineurin/nuclear factor of activated T (NFAT) pathways [3]. Cytokines modulate T-Cell differentiation, function, and proliferation, and this is regulated primarily by the Janus kinase/signal transducer and activator of the transcription protein (JAK/STAT) signaling pathway [4]. 

TCR gamma/delta T-lymphocytes also develop in the thymus but only comprise ~5% of the T-Cell repertoire [5]. Unlike TCR alpha/beta T-lymphocytes, they do not require MHC class I or II antigen presentations but are innate immune cells mostly located in the spleen, skin, and the gut, serving as the first line of defense against foreign peptides [5].

Gene expression profiling and next-generation sequencing of peripheral T-Cell lymphomas have shown that disruption of normal development and differentiation can occur in any of the different signaling pathways related to TCR signaling, co-stimulatory signaling, and cytokine signaling. It occurs mostly through point mutations and copy number changes, leading to the constitutive activation of pathways downstream of the TCR, co-stimulatory molecules, and/or cytokine receptors [6]. 

**Figure 1:**
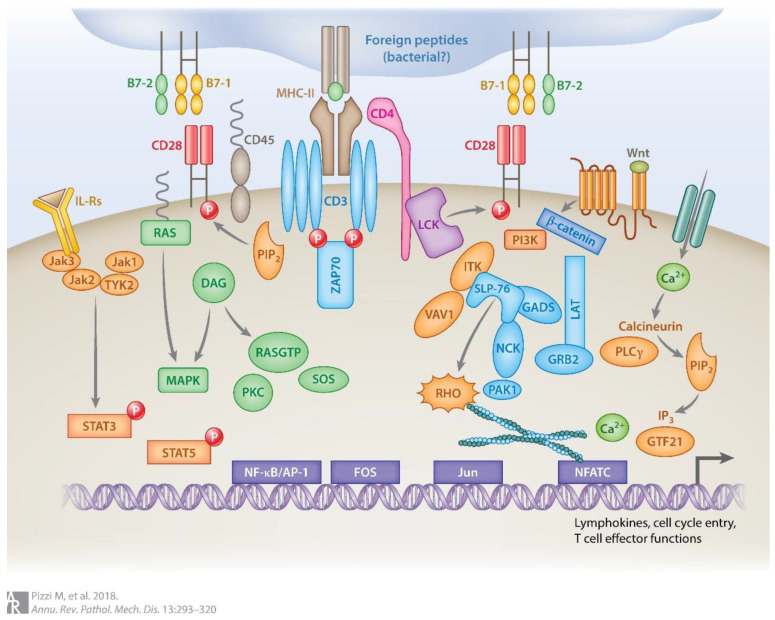
The receptors and signaling pathway components are depicted in this image and explained in detail in the body of the text. For abbreviations, please see the abbreviation list.

Within the TCR signaling pathways, TCR-associated kinases and TCR pathway phosphatases control all the significant events associated with appropriate T-Cell activation. The recruitment of SRC family kinases (SFKs) LCK and FYN, upon binding of the TCR to the MHC complex, initiates a cascade of activating phosphorylation involving ZAP-70, LAT, VAV1, Tec family tyrosine kinase ITK, SYK, and phospholipase C gamma (PLCG1), among many others [3]. VAV1 activates RHO family members RAC1 and RHOA, which maintain a functioning cytoskeleton and activate the phosphatidylinositol-3 protein kinase (PI3K) pathway—a pathway critical for T-Cell differentiation [3]. PLCG1 activates the MAPK pathway, which regulates T-Cell proliferation, differentiation, apoptosis, and stress responses [3]. Activation of SFKs is not uncommon in T-Cell lymphomas, especially in AITL, PTCL- NOS, and CTCL, and encompasses somatic mutations, copy number alterations or transcriptional dysregulation of genes encoding for the enzymes critical in TCR activation [6]. TCR signaling is summarized in Figure 1 (Reprinted with permission from Ref. [3]. Copyright 2018 Annual Review of Pathology). 

TCR pathway phosphatases, so-called protein tyrosine phosphatase non-receptors (PTPN), oppose the function of SFKs and, therefore, are negative regulators of TCR signaling. DUSP22, for example, inactivates LCK. DUSP22 translocations are relatively specific for ALK-negative anaplastic large cell lymphomas [6].

Co-stimulatory and co-inhibitory signaling guide the degree of T-Cell activation and proliferation. CD28 signaling leads to activation of NFAT, NF-kB and subsequent IL-2 secretion [4,7]. Inducible co-stimulator (ICOS) belongs to the CD28 family and regulates the TFH phenotype. CTLA4 primarily functions by indirectly diminishing signaling through the co-stimulatory receptor CD28, reducing immune responses to weaken self- and tumor antigens [6]. Gene fusions involving ICOS-CD28 and CTLA4-CD28 have been reported in CTCL, AITL, and PTCL-NOS and infer oncogenic properties [6].

Cytokine signaling modulates T-Cell differentiation, function, and proliferation through the JAK family of cytoplasmic tyrosine kinases (JAK1, JAK2, JAK3, and TYK2) and the STAT family proteins [3]. STATs are transcription factors and regulate cell proliferation via cyclin D1 and MYC, among others. In addition to STAT activation, JAK signaling activates MAPK, phosphatidylinositol-3′-kinase (PI3K) and the AKT/mammalian target of rapamycin (mTOR) [7]. Genetic alterations in these transcription factors are found in many types of T-Cell lymphomas, and these are highlighted in Table 1. 

Alterations in the regulator genes of the epigenetic signaling pathway that normally influence gene expression through chromatin modification, such as DNA methylation, histone methylation, or chromatin remodeling without altering the DNA sequence, also contribute to lymphomagenesis [8]. The most common epigenetic alterations in T-Cell lymphoma include the dysregulation of DNA methylation and histone modification through the transfer of acetyl or methyl groups with resultant silencing of tumor suppressor genes and/or expression of proto-oncogenes. DNA methylation within gene regulatory elements is mediated by three members of the DNA methyltransferase (DNMT) family called DNMT1, DNMT3A, and DNMT3B, which execute and maintain methylation of CpG dinucleotides [9]. DNMT3A, TET2, IDH2, and RHOA mutations predominantly impact DNA methylation and are often observed in AITL as well as other T follicular helper lymphomas [9,10]. Histone modifying enzymes (writers, erasers, chromatin remodelers) edit the histone code and have been implicated in the development of many T-Cell lymphomas, including ATLL, CTCL, PTCL-NOS, AITL, HSTL, and ENKTL [9]. Genes implicated in histone modification and chromatin remodeling include: KMT2D and KMT2A (encode H3K4 methyltransferases), KDM6A (encodes H3K27 demethylase), SETD2 (encodes H3K36 methyltransferase and recruits DNMT3B), EP300 and CREBBP (encode H3K18 acetyltransferases) [9].

Disease defining recurrent chimeric gene fusions from translocations, such as NPM/ALK or DUSP22/FRA7H in anaplastic large cell lymphoma is uncommon in mature T-Cell lymphomas. Only a few genetic alterations are restricted to specific subtypes of T-Cell lymphoma, and many cross different T-Cell lymphoma subtypes. Mutual to all types of mature T-Cell lymphomas and present in nearly 90% of cases, is the clonally rearranged TCR gene which is routinely evaluated with a polymerase chain reaction (PCR)-based method to detect TCR gamma gene rearrangements [10]. Although 90% of mature T-Cell lymphomas are alpha/beta TCR-expressing T-Cells, they also carry non-functional TCR gamma gene rearrangements that can be captured by PCR analysis of the TCR gamma genes. In general, TCR alpha and beta loci are too complex for routine DNA clonality analysis [10]. 

The following paragraphs will discuss the pertinent genetic aberrancies in the peripheral T-Cell lymphoma subtype, which are summarized in Table 1.

## 2. Mature T-Cell Lymphomas with Leukemic Presentation

### 2.1. T-Cell Prolymphocytic Leukemia

Of the mature T-Cell lymphomas with a leukemic presentation, T-Cell prolymphocytic leukemia (T-PLL) shows the most consistent recurrent genetic alterations. These consist of inv(14)(q11q32) or t(14;14)(q11;q32) juxtaposing the TCR alpha/beta locus at 14q11 to the oncogenes TCL1A or TCL1B at 14q32.1, or to the oncogene MTCP1 at Xq28 in t(X;14)(q28;q11). These comprise >90% of T-PLL cases [11]. The second most common cytogenetic abnormality involves chromosome 8 (70–80% of cases), including isochromosome 8q and trisomy 8, resulting in an increased copy number of MYC at 8q24.2 [10]. MYC regulates cell growth, proliferation, and induction of telomerase activity [12]. MYC activation has been implicated in various lymphoid neoplasms and is found in 67% of T-PLL cases studied by Hsi et al. [13]. Other recurrent chromosomal aberrations in T-PLL include 11q23 deletion resulting in loss or dysregulation of the ataxia telangiectasia mutated (ATM) gene, chromosome 17 aberrations leading to TP53 gene deletion at 17p13.1 and deletions of 5q, 6q, 7q, 11q, 12p, 13q, 17p and 20q [12]. Both ATM and TP53 genes are tumor suppressor genes playing essential roles in cell cycle arrest, DNA repair, and apoptosis upon DNA double-strand breaks [14]. Despite the numerical and structural abnormalities in multiple chromosomes seen in T-PLL, no individual abnormality correlates with prognosis [12]. 

Mutations also frequently affect the ATM and TP53 genes, causing their inactivation. ATM encodes a nuclear phosphoprotein in the phosphatidylinositol-3 protein kinase (PI3K) family, regulating the cell cycle and DNA repair. Patients with ataxia telangiectasia harboring ATM germline mutations are susceptible to developing T-PLL [11]. Missense mutations in the ATM locus at 11q23 are most common (73%), followed by frameshift mutations in 16% of cases [14]. Similarly, most mutations in TP53 represent missense mutations, as well (71%) [14]. Whole exome and targeted sequencing of T-PLL have also shown gain-of-function mutations involving genes in the JAK/STAT signaling pathway. Mutations of JAK3 have been documented in 30–42% of cases, JAK1 in 8% and STAT5B in 21–36% [15]. Thus far, JAK3 mutations are the only genetic alteration with prognostic significance in T-PLL associated with adverse outcomes [11]. 

### 2.2. T-Cell Large Granular Lymphocytic Leukemia

Gain-of-function mutations in STAT3 and STAT5B genes are the most frequent abnormalities in T-Cell large granular lymphocyte leukemia (T-LGLL) [16]. Activating STAT3 mutations are typically seen in the most common type of T-LGLL, the CD8+ T-LGL type, with an incidence ranging from 11–75% based on different reports [16]. These are preferentially located in the Src homology 2 (SH2) domain of the gene, leading to increased stability of STAT3 protein dimerization and enhanced transcriptional activity of pro-survival proteins [16]. Many studies have revealed that a STAT3 mutation alone is unlikely to initiate clonal proliferation of T-LGL [16]. In vitro inhibition of STAT3 restored large granulocyte lymphocyte apoptosis regardless of STAT3 mutational status [16]. Additionally, murine cells transduced with retrovirus showed that STAT3 mutants do not provide any cell growth advantage, and mouse models have shown that a STAT3 mutation alone is insufficient to induce T-LGLL [16]. These findings suggest that additional gene mutations or dysregulation of other signaling pathways associated with STAT3 mutations contribute to pathogenesis [16]. STAT5B mutations primarily involve CD4+ T-LGLL (15–55%), which represents a rare variant. These mutations are very rare in CD8+ T-LGLL, and when present, appear to confer a poor prognosis compared to CD4+ T-LGLL harboring these mutations [16]. The IL-15-STAT5 axis has been postulated to contribute to lymphomagenesis, as IL-15 is a proinflammatory cytokine upstream of STAT5B, and transgenic mice overexpressing IL-15 develop aggressive variants of T or NK-cell leukemia [16,17]. The indolent course of CD4+ T-LGLL with STAT5B mutations suggest these disorders lack one or more concurring events with STAT5B [16].

### 2.3. Adult T-Cell Leukemia/Lymphoma

Adult T-Cell leukemia/lymphoma (ATLL) is a rare peripheral T-Cell neoplasm with virus-mediated oncogenesis where a clonally integrated human retrovirus human T-Cell leukemia virus type 1 (HTLV-1) is found in all cases. It is endemic in Japanese, Caribbean, and Latin American populations. Although HTLV-1 is causally linked to ATLL, the long latency period indicates HTLV-1 infection alone is insufficient to induce neoplastic transformation. Only 2.5% of infected individuals are at risk of developing ATLL [15,18]. ATLL shows frequent gain-of-function alterations in the TCR/nuclear factor kappa-light-chain-enhancer of the activated B-cells (NF-kB) signaling pathway [19]. Under normal circumstances, NF-kB is critical for the induction of inflammatory responses in T-Cells, survival, and differentiation. The T-Cell receptor and co-receptor CD28 are the central regulators of NF-kB activation in T-Cells. Within the TCR/NF-kB signaling pathway, the most commonly altered gene is PLGC1 (36%) encoding for phospholipase C λ-1, essential for the downstream nuclear factor of activated T-Cells (NFAT) and NF-kB activation [20]. Furthermore, ~7% of ATCL harbor CTLA4-CD28 and ICOS-CD28 fusion genes, leading to prolonged CD28 co-stimulatory signaling and T-Cell proliferation [19]. In general, aggressive subtypes have higher frequencies of TP53 and IRF4 mutations, as well as copy number alterations (CNAs), including PD-L1 amplifications and CDKN2A deletions [20]. In contrast, STAT3 mutations are associated with indolent ATLL [20]. Given the indolent behavior of STAT3 mutations in T-Cell large granulocytic leukemia and, also chronic lymphoproliferative disorders of NK-cells (not discussed here), this suggests that STAT3 mutations are characterized by a slow progressive expansion of clonal mature T- and NK-cells [20].

## 3. Extranodal T-Cell Lymphomas

### 3.1. Hepatosplenic T-Cell Lymphoma

Of the extranodal T-Cell lymphomas, hepatosplenic T-Cell lymphoma (HSTL) shows a relatively specific recurrent genetic aberrancy. Neoplastic T-Cells are of TCR gamma/delta type, and the most common genetic abnormalities include isochromosome 7q (63%) and trisomy 8 (50%), which can co-occur [21]. Ring chromosome 7 leading to 7q amplification, loss of Y chromosome, loss of chromosome 10q (19%) and gain of chromosome 1 (13%) have also been reported [21]. 

Mutations in epigenetic modifier genes are also common. McKinney et al. studied 68 cases of HSTL, showing that chromatin remodeling genes were the most frequently mutated group (62% of cases), particularly SETD2 (25% of cases) [22]. The majority of SETD2 alterations encompass nonsense and frameshift mutations that cause loss-of-function. The SETD2 gene encodes for histone lysine methyltransferase, which belongs to the KMT1 family that regulates heterochromatin–nuclear periphery tethering via histone and non-histone protein methylation. McKinney et al. postulate that SETD2 acts as a suppressor gene with loss-of-function and increased proliferation of lymphoma cells. Other frequently mutated chromatin modifier genes include INO80 (21%), TET3 (15%) and SMARCA2 (10%) [22]. Missense mutations in STAT5B, STAT3, and PIK3CD have also been reported and, similar to other T-Cell lymphomas, also occur in the SRC homology 2 domains, causing constitutive activation of the proteins [21,22]. STAT5B and STAT3 mutations are virtually exclusive in occurrence, whereas STAT5B and PIK3CD mutations potentially complement each other in creating cooperativity between PI3K and JAK-STAT signaling in maintaining proliferation pathways in HSTL lymphoma cells.

### 3.2. Intestinal T-Cell Lymphomas

Intestinal T-Cell lymphomas are categorized as enteropathy-associated T-Cell lymphoma (EATL), monomorphic epitheliotropic intestinal T-Cell lymphoma (MEITL), intestinal T-Cell lymphoma, not otherwise specified (NOS), and indolent T-Cell lymphoproliferative disorder of the gastrointestinal tract (ITLPD-GIT) [15]. The latter, ITLPD-GIT, is a heterogeneous group of clonal T-Cell diseases that have only recently been sufficiently characterized to become a provisional entity in the revised 4th edition of the World Health Organization (WHO) classification of lymphoid neoplasms, but genetic aberrancies in this subcategory continue to be limited.

EATL is associated with celiac disease and primarily affects individuals of northern European descent, while MEITL shows an increased incidence in Asian and Hispanic populations without association with celiac disease [15]. There is a considerable overlap of genetic aberrancies between EATL and MEITL. Genomic aberrations involving 9q34.3 gains are the most common changes seen in both EATL (80%) and MEITL (75%) and can be identified by fluorescence in situ hybridization and copy-number analysis [15]. Loss of 16q12.1 is also seen in both types, whereas gains of 1q and 5q are characteristic of EATL and gains of 8q with amplification at the MYC locus are characteristic of MEITL [23]. Whole exome sequencing of both types generally shows the JAK/STAT pathway to be the most frequently mutated pathway with frequent mutations in the STAT5B, JAK1, JAK3, and STAT3. STAT5B mutations occur more frequently in MEILT. Additionally, mutations of the epigenetic SETD2 gene which encodes for histone lysine methyltransferase have been reported in >90% of MEITL [23]. 

### 3.3. Epstein–Barr Virus Positive T-Cell and Natural Killer-Cell Lymphoproliferative Disorders

The 2016 revised WHO classification of mature T- and NK-cell neoplasms recognizes eight different categories of Epstein–Barr virus (EBV) positive T-Cell and natural killer (NK) cell lymphoproliferative disorders (LPD), depending on age group and localized (mostly skin) or systemic manifestation. 

EBV-mediated oncogenesis is implicated in all categories. EBV is a double-stranded DNA gamma herpesvirus detected in up to 95% of the population worldwide [24]. EBV has a tropism for B cells but can infect other types of immune cells, including T-Cells, NK-cells, and epithelial cells [25]. Primary EBV infections are often clinically silent and lifelong latent infections occur without complications [24]. However, EBV primary infection and secondary reactivation may lead to EBV-associated lymphoproliferative disorders [24]. Primary infection occurs through oral transmission of oropharyngeal epithelial cells, where the virus undergoes replication and expresses its DNA genome to create new viral particles [24]. EBV then infects naïve B cells and establishes latent infection using memory B cells as a reservoir [24]. There are three types of latency programs, classified by their expression of EBV latency molecular markers (EBNA1, EBNA2, EBNA3A, EBNA3B, EBNA3C, and EBNA-LP), latent membrane proteins (LMP1, LMP2a, LMP2B) and non-coding RNAs (EBER1, EBER2) [24,26]. EBV latent antigens are implicated in the development of B-cell lymphomas and EBV-associated T/NK lymphoproliferative disorders [24]. Expression of all six nuclear proteins (EBNAs) and all three cell membrane-associated proteins (LMPs) is referred to as type III latency [24,26]. Cells with type III latency only exist during acute primary EBV infection (prior to EBV-specific T-Cell response) and in immunocompromised patients [26]. Type II latency, lacking EBNA2 expression, allows infected cells to survive and escape immune surveillance by restricting latent gene transcription mechanisms [24]. Type I latency, involved in the maintenance and replication of the episomal viral genome, expresses EBNA1 and EBERs [24]. 

In contrast to the well described mechanisms leading to EBV infection of B cells, the mechanism of T/NK-cell infection by EBV remains unclear [24]. While host immune dysregulation and EBV replication may incite EBV-positive lymphoproliferative disorders, other hypotheses involve CD21, the primary receptor for EBV [27]. It is hypothesized that NK-cells can attack CD21+ EBV-infected B cells and acquire weak CD21 expression by synaptic transfer of receptor molecules onto their own membrane, permitting EBV binding [24,27]. There is also speculation that CD21 may be expressed on immature T-Cells or early lymphocyte progenitors [24]. 

Four of the eight EBV-positive T-Cell and NK-cell lymphoproliferative disorders are overtly malignant, and these are extranodal NK/T-Cell lymphoma, nasal type, aggressive NK-cell leukemia systemic EBV-positive T-Cell lymphoma of childhood, and primary EBV-positive nodal T/NK-cell lymphoma, which represents a provisional entity [15]. Among these, the most extensively studied represents extranodal NK/T-Cell lymphoma (ENKTL), nasal type, which typically arises in middle-aged adults from Asia and Latin America with a predilection for the nasal cavity, and is less common for skin, gastrointestinal tract, and the testes [15]. In most cases, lymphoma cells are derived from NK-cells, and therefore will not show a clonally rearranged TCR gene. The role of EBV in oncogenesis is not entirely clear, but it is believed that LMP1 may induce pro- and anti-apoptotic signaling of NK-cells through NF-kB signaling activation and microRNA deregulation [24]. Many studies have also shown upregulation of PD-ligand 1 (PD-L1), induced by anti-inflammatory cytokines, the activation of the STAT3 pathway and LMP overexpression [24]. PD-L1 overexpression contributes to NK cell growth by helping neoplastic cells escape immune surveillance [24]. A more common cytogenetic abnormality is 6q16–27 deletion, resulting in loss of tumor suppressor genes, such as PRDM1 or ATG5. Others include del(6)(q21q25) and the loss of 2q and 1p36.23–36.33 [15]. Next-generation sequencing of ENKTL and aggressive NK-cell leukemia (ANKL) has shown recurrent mutations in the JAK/STAT pathway, particularly in STAT3 and STAT5B in both lymphoma entities. Unlike ENKTL, ANKL has not shown JAK3 mutations, thus far, but case numbers are limited [24]. More recently, alterations in epigenetic regulators (KMT2D, KMT2C, ASXL3, ARID1A, EP300) have been detected [24]. Systemic EBV-positive T-Cell lymphoma of childhood (STCLC) is a life-threatening disease for children and young adults, and occurs shortly after primary EBV infection [15]. The etiology is unknown, but the association with EBV and predominance in Asia suggests a genetic defect in the host’s response to EBV [15]. Most cases show monoclonal TCR rearrangements and EBV in a clonal episomal form. Targeted next-generation sequencing in a cohort of 169 EBV-associated T/NK LPD, including 34 STCLC cases, showed mutations of the epigenetic gene KMT2D to be most frequent (17% of cases) [24].

## 4. Cutaneous T-Cell Lymphomas

### Mycosis Fungoides/Sezary Syndrome

Mycosis fungoides (MF)/Sezary syndrome (SS) represent 50–60% of cutaneous T-Cell lymphomas [15]. No disease defining genetic abnormalities exist, but recent studies have shown that oncogenic aberrations appear to affect genes in the JAK/STAT and TCR/NF-kB signaling pathways [28]. Alterations of epigenetic regulators and of genes regulating cell cycle and DNA repair have also been reported. Copy number gains of STAT3 (~60%), STAT5B (~60%), and JAK2 (~13%) are more common than point mutations which occur mostly in STAT5B (~3.6%) [28]. Point mutations in the PLGC1 gene encoding for phospholipase C λ-1 (10% of cases) and point mutations in CD28 (3.6%), as well as CTLA4-CD28 and ICOS-CD28 fusion genes in rare cases, contribute to altered TCR/NF-kB signaling. Epigenetic alterations in MF/SS predominantly affect DNA methylation. Under normal circumstances, DNA methylation maintains chromosomal stability, whereas low methylation allows for transcription at gene promotors. Loss-of-function alterations in DNA methyltransferases can lead to hypomethylation promoting genome instability and transcriptional dysregulation. In SS, the DNA methyltransferase DNMT3A is most frequently deleted (38%), but point mutations in the gene encoding for this enzyme also occur in ~4% of cases. Additionally, alterations in chromatin remodeling leading to the disruption of normal gene expression are also frequent. For example, deletion of AT-Rich Interaction Domain 1A (ARID1A) and ARID5B have been reported in ~58% and ~29% of SS cases, respectively, and point mutations in ARID2 have been reported in a subset of MF cases [28].

Lastly, genes encoding for proteins that are indirectly involved in the regulation of the cell cycle, that are altered in SS, involve deletion of tumor suppressors TP53 (~93%), CDKN2A (~40%), RB1 (~39%), ATM (~30%), and CDKN1A (~11%) [28]. Tumor suppressors control cell proliferation, activate apoptosis to eliminate unwanted cells, link DNA damage signals to cell cycle arrest checkpoints, activate DNA repair and inhibit metastasis by preventing loss of cellular adhesion molecules [29].

## 5. T-Cell Lymphomas with T Follicular Helper Cell Origin, including Angioimmunoblastic T-Cell Lymphoma, Follicular T-Cell Lymphoma, and Nodal Peripheral T-Cell Lymphoma with TFH Phenotype

It is well recognized that TFH cells preferably reside in germinal centers where they provide critical T-Cell help to germinal center B-cells for germinal center formation, affinity maturation, and development of high-affinity antibodies [30]. Principally, T-Cell lymphomas with TFH cell origin show an over-representation of mutations in the epigenetic regulator genes, the TCR and co-stimulatory signaling genes, and the ras homology family member A (RHOA) genes [3]. The most common mutations in the epigenetic regulator genes are loss-of-function mutations in the Ten-Eleven Translocation 2 (TET2) gene, which were first identified in myeloid neoplasms [31]. They occur in as many as 80% of angioimmunoblastic T-Cell lymphoma (AITL) cases and 58% of T follicular helper (TFH)-like PTCL [30,32]. The gene encodes for the DNA demethylation enzyme TET2 involved in promoting DNA demethylation, which is critical in regulating gene expression [30,33]. The enzyme appears to act as a tumor suppressor. TET2 mutations are predominantly insertions and deletions generating frameshift and nonsense mutations, similar to those found in myeloid neoplasms [33]. TET2 mutations have been shown to decrease T regulatory cells by destabilizing FOXP3 [34]. Interestingly, laser capture microdissected bystander B-cells in AITL cases were found to harbor TET2 mutations, as well, supporting the presence of co-existing oligo-or monoclonal B-cell proliferations in AITL and concurrent B-cell lymphoma development in a subset of AITL patients [35]. The second most common epigenetic regulator mutation represents the isocitrate dehydrogenase (IDH) 2 mutations located at the p.R172 position [36,37]. It confers oncogenic potential by an enzymatic gain-of-function due to conversion of 2-oxoglutarate to abnormally high levels of D-2-hydroxyglutarate (D-2-HG) which acts as an oncometabolite [31]. This mutation is exclusively found in T-Cell lymphomas with TFH origin and occurs in ~20% of AITL [3]. The coexistence of IDH2 and TET2 mutations in ~20% of AITL cases suggests a supportive relationship [7,31,33]. It has been proposed that D-2-HG contributes to the functional inactivation of the TET2 protein. TET2 mutations also coexist with DNA methyl transferase 3 alpha (DNMT3A) gene mutations [7,31]. The most common DNMT3A gene mutation is a point mutation R882H causing aberrant DNA methylation, and it occurs in roughly 30% of AITL. The DNMT3A R882H has been shown to contribute to TET2 inactivation. In murine models, TET2 inactivation has been linked to TFH cell overproduction and the development of T-Cell lymphoma with a TFH phenotype [31].

Approximately 13% of T-Cell lymphomas with a TFH origin show point mutations and translocations involving CD28, representing the most common genetic alteration in the TCR signaling pathway [4,31]. In general, most TCR signaling mutations are gain-of-function mutations with downstream activation of the NFAT, NF-kB, and MAPK pathways. In addition, genetic alterations leading to altered expression of CD28 have been shown to provide a non-neoplastic microenvironment suitable for AITL development and uncontrolled lymphoma proliferation through immunosurveillance evasion.

A normal TCR and co-stimulatory signaling pathway require a functional T-Cell cytoskeleton which is regulated by small guanosine triphosphate (GTP)-binding proteins, such as Rho GTPases (RhoA, RhoB, and RhoC). An inactivating mutation of the RHOA gene encoding a p.Gly17Val is present in ~70% of AITL, whereas it is present in only 18% of other T-Cell lymphomas with a TFH phenotype and is very rare in other peripheral T-Cell lymphomas [4,38]. The resulting RhoA G17V protein is impaired in GTP binding, which in murine models has shown to lead to increased numbers of TFH cells [39]. Finally, Sakata-Yanagimoto et al. have also found that mutations in RHOA, TET2, and IDH2 occur in combinations. In their study, all RHOA mutated cases also showed TET2 mutations, and the vast majority of IDH2 mutations co-existed with RHOA and TET2 mutations [38]. The mutant TET2 alleles show a significantly higher allelic burden compared to the other two mutant genes, and this suggests that TET2 mutations precede RHOA and/or IDH2 mutations [38].

Of T-Cell lymphomas with T follicular helper cell (TFH) origin, follicular T-Cell lymphoma has been associated with the t(5;9)(q33;q22) that fuses ITK to SYK, resulting in overexpression of the SYK tyrosine kinase [40]. SYK and ZAP-70 are structurally similar and are involved in initiating multiple signaling cascades, including MAPK signaling and cytokine secretion. The translocation appears to be relatively specific for this entity and occurs in ~20% of cases [40].

## 6. Genetic Drivers in ALK-Negative Anaplastic Large Cell Lymphomas (ALCL) and Their Role in Differentiating These from CD30+ Peripheral T-Cell Lymphoma, Not Otherwise Specified (NOS) and Primary Cutaneous Anaplastic Large Cell lymphoma

Although DNA methylation profiling has shown a possible thymic origin for both ALK-positive and ALK-negative anaplastic large cell lymphoma (ALCL), suggesting a common cellular origin, driver genetic alterations have recently emerged in ALK-negative ALCL that are distinct from ALK-positive ALCL [41]. Furthermore, 30% of ALK-negative ALCL shows a rearrangement of DUSP22 on 6p25.3, a gene encoding the dual-specificity phosphatase 22 that inhibits T-Cell receptor signaling [42]. This translocation leads to decreased expression of DUSP22. The most common translocation partner is the FRA7H fragile site on 7q32.3 [42]. Interestingly, this translocation is also present in ~20–30% of primary cutaneous ALCL, and largely absent in CD30+ peripheral T-Cell lymphoma, not otherwise specified (PTCL-NOS) [41,43]. Another discrete genetic aberration in ALK-negative ALCL, although only occurring in 8% of cases, is TP63 rearrangements, most frequently with TBL1XR1 as a result of inv(3)(q26q28) [44]. Moreso, p63 is a member of the p53 family and is involved in the regulation of cell cycle arrest, apoptosis, and tumorigenesis. This translocation has also been found in CD30+ PTCL-NOS and is rare in primary cutaneous ALCL. Both the DUSP22 and TP63 gene rearrangements can be detected by fluorescence in situ hybridization and are mutually exclusive. Since both gene rearrangements are not exclusive to the diagnosis of ALK-negative ALCL, in contrast to ALK rearrangements in ALK-positive ALCL, the presence of these thus support but do not confirm the diagnosis and should be interpreted in the context of morphologic, immunophenotypic and clinical findings. Regarding prognosis, ALK-negative ALCL patients with a DUSP22 rearrangement have a similarly favorable outcome as ALK-positive ALCL patients, whereas TP63 rearranged ALK-negative ALCL patients usually display an aggressive clinical course [45,46,47]. 

Numerous studies have attempted to genetically discriminate between ALK-negative ALCL and CD30+ PTCL. Agnelli and al. identified a 3-gene model of CD30/TNFRSF8, BATF3, and TMOD1 genes that separated ALK-negative ALCL from CD30+ PTCL with a 97% accuracy. These genes overexpressed in ALK-negative ALCL compared to CD30+ PTCL [48].

While STAT3 activation is induced by ALK fusion proteins in ALK-positive ALCL, it is induced by somatic mutations of STAT3 and JAK1 in 20% of ALK-negative anaplastic large cell lymphomas and 5% of primary cutaneous ALCL, whereas mutations in these genes have not been reported for CD30+ PTCL [41,49,50]. Instead, JAK2 rearrangements, most commonly with the PCM1 gene as the fusion partner, appear to derange the JAK/STAT pathway in peripheral CD30+ PTCL [51]. Besides mutations in STAT3 and JAK1, oncogenic fusion genes, such as NFKB2-ROS1 and NFKB2-TYK2, also contribute to STAT3 activation in ALK-negative ALCL [49]. Furthermore, 24% of ALK-negative ALCL shows aberrant levels of truncated Erb-B2 receptor tyrosine kinase 4 (ERBB4) transcripts [52]. ERBB4 gene encodes for a member of the tyrosine kinase receptor superfamily and is a member of the epidermal growth factor receptor (EGFR) subfamily.

Lastly, chromosomal imbalances in primary cutaneous ALCL include losses of 3p, 6q16–6q21, and 8p, and gains in 7q31, 17q, and 21 [41].

## 7. Genetic Pathways in Peripheral T-Cell Lymphoma, Not Otherwise Specified

Peripheral T-Cell lymphoma, NOS, encompasses a heterogenous group of mature T-Cell lymphomas with broad morphologic and immunophenotypic features that do not match any of the specifically defined entities of mature T-Cell lymphomas in the current WHO classification. These generally confer a poor clinical outcome. Gene expression profiling delineated two major molecular subgroups within this group based on the high expression of transcriptional regulators, GATA3 and TBX21, and its target genes. GATA3 is the master regulator in T-helper 2 (TH2) cell differentiation and regulates IL-4, Il-5, and IL-13 expression, whereas TBX21 is the master regulator of TH1 and cytotoxic T-Cell differentiation and regulates interferon-gamma and granzyme B. Based on this differentiation, two distinct peripheral T-Cell lymphoma groups, PTCL-GATA3 and PTCL-TBX21, have emerged [53]. PTCL-GATA3 appears to be associated with a loss or mutations of tumor suppressor genes in the CDKN2A/B-TP53 pathway and PI3K pathways, as well as genetic gains and amplifications of STAT3 and MYC. PTCL-TBX21 appear to show mutations in genes regulating DNA methylation, such as in TET1, TET3, and DNMT3A, however, are still less frequent in this subgroup than in angioimmunoblastic T-Cell lymphoma [54,55]. Furthermore, PTCL-TBX21 is associated with gene aberrancies, leading to NF-kB activation [53].

## 8. Biomarkers as Therapeutic Targets for Peripheral T-Cell Lymphomas

With the recent advances in understanding the pathogenesis and molecular pathway of peripheral T-Cell lymphoma, targeted molecular therapy and immunotherapy increase treatment options beyond the traditional cyclophosphamide, doxorubicin hydrochloride, vincristine sulfate, and prednisone (CHOP) and CHOP-like chemotherapies. These emerging therapy options encompass antibodies, histone deacetylase inhibitors (HDACi), chimeric antigen receptor T-Cells (CARTs), immune checkpoint inhibitors, and inhibitors targeting the TCR/co-stimulatory and JAK/STAT pathways used alone or in combination. Clinical trials regarding these therapies appear to preferentially involve AITL, ALCL, PTCL-NOS, and cutaneous T-Cell lymphoma. 

### 8.1. Antibody-Based Therapies

Of cell surface targets in peripheral T-Cell lymphoma, CD30 appears particularly promising, especially for ALCL, regardless of ALK-1 expression. It is a transmembrane protein belonging to the tumor necrosis factor receptor (TNFR) superfamily, and normal expression is restricted to a very small subset of activated B, T, and NK-cells. The exact pathways involved in CD30 signaling and its role in cytokine production, receptor expression, T-Cell proliferation or induction of apoptosis are not well understood [56]. Furthermore, the underlying genetic alterations leading to disproportionally strong CD30 expression in ALCL are not clear, as well. The CD30 receptor is a key modulator of T-Cell activity, and brentuximab vedotin (BV) is an antibody linked to the anti-tubulin agent monomethyl auristatin E (MMAE), causing disruption of the microtubule network on lymphoma cells and subsequent cell cycle arrest and apoptosis [57]. BV has thus far been approved for the treatment of classical Hodgkin lymphoma, primary cutaneous and systemic ALCL, AITL, PTCL-NOS, and MF/SS. Recently published data on the addition of BV to traditional CHOP in untreated patients with CD30+ PTCL are very promising, in that this combination resulted in higher rates of progression-free survival (PFS) than that of CHOP alone (48 vs. 21 months) [58].

The surface CD52, also known as the CAMPATH-1 antigen, can also be targeted since this antigen is widely expressed on peripheral T-Cell lymphomas. It is a peptide of 12 amino acids anchored to glycosylphosphatidylinositol (GPI), and its exact function remains unknown; however, it appears to play a role in lymphocyte production and production of TNF-a, IFN gamma, and IL-6 [59]. Alemtuzumab induces complement-mediated lysis and apoptosis in lymphoma cells and has been tested as a sole agent in clinical trials involving patients with heavily pretreated and refractory PTCL and MF/SS [57]. More recently, alemtuzumab is being tested in a clinical trial in combination with IL-15 in patients with relapsed ATLL [60].

High levels of CD25 expression are particularly found in AITL, ATLL, and ALCL. CD25 represents the alpha chain of the interleukin-2 (IL-2) receptor. Its upregulation has been shown to promote lymphomagenesis and drug resistance [61]. Basiliximab is a chimeric mouse/human antibody that specifically binds and blocks the alpha chain of the IL-2 receptor, leading to competitive inhibition of IL-2 binding and consequent inhibition of T-Cell proliferation and activation. Radioimmunotherapy with conjugation of the chimeric basiliximab to iodine-131 has shown promise in patients with refractory T-Cell lymphoma. Therapy was well-tolerated and of the nine patients enrolled, three patients showed a complete and three patients a partial response [62]. Camidanlumab tesirine (ADCT-301), where the CD25 antibody is conjugated to cytotoxic pyrrolobenzodiazipine (PBD), has also been tested in clinical trials. Its mechanism of action relates to cell death by cross-linkage to DNA and blockage of DNA replication. Early results show antitumoral activity in both classical Hodgkin lymphoma and non-Hodgkin lymphoma, including PTCL [63].

The anti-CD38 antibody daratumumab is a human IgGκ monoclonal antibody targeting CD38 with a direct on-tumor and immunomodulatory mechanism of action. Zaja et al. demonstrated that ~80% of AITL and 60% of PTCL-NOS express variable levels of CD38, and it is also expressed on most ENKTL [64]. Binding to CD38 causes tumor cell death mediated by both the antibody-dependent cellular phagocytosis (ADCP), where CD32A induces the phagocytosis of the tumor cell, and antibody-dependent cell-mediated cytotoxicity (ADCC), where CD16A induces the release of cytotoxic granules to kill the tumor cell. It has shown an overall response rate of 25% in patients with relapsed or refractory NK/T-Cell lymphomas and is currently being investigated in combination with gemcitabine, cisplatin, and dexamethasone in patients with PTCL-NOS and T-Cell lymphomas with TFH immunophenotype [65].

Chemokine receptor 4 (CCR4) is expressed at variable levels on ATLL, ALK-negative ALCL (~65%), PTCL-NOS, AITL, and transformed MF (20–40%). Mogamulizumab has a direct cytotoxic effect by antibody-dependent cellular cytotoxicity (ADCC) and by depletion of regulatory T-Cells, which leads to enhanced anti-tumor immunity [57]. It revealed an overall response rate of ~35% in patients with relapsed PTCL and CTCL [66].

### 8.2. Chimeric Antigen Receptor T-Cells (CARTs)

CARs are engineered synthetic receptors that function to redirect T-Cells to recognize and eliminate cells expressing a specific target antigen. CAR binding to target antigens expressed on the cell surface of the cancer cell is independent of the MHC receptor, resulting in vigorous T-Cell activation and powerful anti-tumor responses. Finding a suitable target antigen that the CARs can be directed to is challenging since most T-Cell associated antigens are the same for neoplastic and normal cells. In treatment for T-Cell lymphomas, chimeric antigen receptors have been engineered targeting CD7, CD4, CD5, CD30, and TCR [60,67]. Overall, the development of CAR-T therapy for mature T-Cell malignancies has been hampered by the absence of target antigens that are preferentially present on neoplastic T-Cells, and not on the normal counterpart cell. Furthermore, some T-Cell associated antigens, especially CD7, may not serve as a target, since their expression may be aberrantly diminished or absent in neoplastic T-Cells. Moreso, CARTs express these T-Cell associated antigens themselves, becoming a target for cell destruction. Lastly, the purity of harvested autologous T-lymphocytes for cellular engineering and culturing and subsequent infusion may be compromised due to contamination b residual neoplastic T-Cells. Current clinical trials of CARTs for T-Cell lymphoma are early studies that mainly address safety, pharmacokinetics, and the overall clinical response [60,67].

### 8.3. Immune Checkpoint Inhibitors 

Immune checkpoint inhibitors (ICIs) are immunoregulatory antibodies promoting host immune responses and subsequent cytotoxicity. The most studied targets for checkpoint inhibition are cytotoxic T-lymphocyte associated protein 4 (CTLA4) and programmed death-receptor 1 (PD-1) or -ligand 1 (PD-L1). CTLA4 primarily functions by indirectly diminishing signaling through the co-stimulatory receptor CD28, reducing immune responses to weaken self- and tumor antigens. PD-1 binds to PD-L1, causing inhibition of T-Cell proliferation, survival, and cytokine release. PD-L1 is frequently expressed in AITL and PTCL with TFH phenotype, and therefore may be a promising therapy option for patients with these subtypes. Clinical trials with anti-PD-1 (nivolumab, pembrolizumab) or anti-PD-L1 (atezolizumab, avelumab, durvalumab) for T-Cell lymphomas are small and show no clear benefit. However, pembrolizumab for advanced Sezary syndrome showed a promising overall response rate of 38% with substantial improvement of the skin lesions [68]. CTLA4-CD28 gene fusions have been preferably observed in AITL, PTCL-NOS, ATLL, and cutaneous T-Cell lymphomas and, therefore, may represent a potential target for anti-CTLA4 immunotherapy. Clinical trials on the efficacy in T-Cell lymphoma are rare, but one case report reports a rapid clinical response with the CTLA4 inhibitor ipilimumab in a patient with Sezary syndrome harboring a highly expressed gene fusion between CTLA4 and CD28 [69].

### 8.4. Targeting Epigenetic Regulators 

Epigenetic dysregulation plays a major role in AITL, PTCL-NOS, CTCL, and extranodal NK/T-Cell lymphoma (ENKTL) [57]. Molecular inhibitors targeting epigenetic mechanisms include histone deacetylase inhibitors (HDACi), such as vorinostat or belinostat that induce apoptosis and cell cycle arrest through the accumulation of acetylated histones. Normal deacetylation of lysine residues in chromatin is prevented [57]. Vorinostat was first approved in 2006 for treatment in CTCL patients with progressive disease. Belinostat was first approved for the treatment of refractory PTCL in 2014, and it showed an overall response rate of 25% in patients with PTCL-NOS, and AITL [70]. In fact, a recent study showed that HDACi leads to a better response in PTCL with T follicular helper phenotype than in PTCL without T follicular helper phenotype [71]. However, overall, the response of HDACi in the treatment of T-Cell lymphoma is only 30%. Therefore, many clinical trials have been conducted to test HDACi in conjunction with other drugs or radiation. Furthermore, HDACi is also being incorporated into hematopoietic stem cell transplantation (HCT). One promising combination represents romidepsin and doxorubicin for relapsed/refractory CTCL and PTCL, where in a phase-1 study, the overall response rate in CTCL patients was 70% [72]. 

Furthermore, 5-Azacytidine is an analog of the nucleoside cytidine which functions as a hypomethylating agent by incorporating into DNA where it irreversibly binds to DNA methyltransferases and therefore inhibits their activity. The response rate to this drug appears to correlate with TET2, IDH1/2, and DNMT3A mutations, and thus, is of particular value for AITL and PTCL-NOS [57]. Sustained responses have been reported in patients with AITL, where 5 of 12 patients showed a sustained response of complete remission > 23 months after treatment initiation [73]. Newer studies show that the combination of 5-azacytidine and the HDACi romidepsin is highly effective in PTCL, showing an overall response rate (ORR) and complete response (CR) of 61% and 48%, respectively. In this multicenter phase II study, patients with PTCL showing a TFH immunophenotype displayed an even better response with an ORR of 80% and CR of 67% [74]. 

### 8.5. Targeting TCR and Co-Stimulatory Signaling Pathways

An important cornerstone of the adaptive immune response is the calcineurin/nuclear factor of activated T (NFAT) pathways leading to T-Cell activation. NFAT comprises five structurally related members: NFATc1, c2, c3, c4, and NFAT5, which are Ca(2+)-regulated transcription factors. They are heavily phosphorylated when in the resting state and are activated through dephosphorylation by calcineurin, where they translocate to the nucleus to activate target gene expression. Cyclosporine A and FK506 are calcineurin inhibitors and block the dephosphorylation of NFAT. They exert their anti-tumor effects by reducing IL-2 production and IL-2 receptor expression, leading to decreased T-Cell activation. The treatment experience with cyclosporine A is promising in AITL with an overall response rate of 86%, reported in a review article of published articles and conference articles regarding this type of treatment [75]. 

TCR signaling can also be targeted with tyrosine inhibitors. For example, p.Gly17Val mutations in the RHOA gene encoding for T-Cell cytoskeleton regulation have been shown to be counteracted by the multikinase inhibitor dasatinib in AITL and PTCL-NOS [76]. Fujisawa et al. demonstrated that the binding of VAV1 to the mutated G17V RHOA accelerates TCR signaling that can, in vitro, be counteracted by dasatinib [77]. Furthermore, the authors identified 8.2 % of VAV1 mutations in 85 RHOA mutation-negative T-Cell lymphoma samples, and the resulting acceleration of mutated VAV1 protein phosphorylation and subsequent accelerated TCR signaling could also be suppressed by dasatinib [77]. It also targets SRC kinase and may be of benefit in PTCL with FYN mutations, where in vitro studies showed dose-dependent inhibition of the increased phosphorylation activity of FYN Leu174Arg, FYN Arg176Cys, and FYN Tyr531His mutant proteins by dasatinib [78].

### 8.6. Targeting the JAK/STAT Pathway

Dysregulated activation of the JAK/STAT pathway appears more common in ALCL, ENKTL, and cutaneous T-Cell lymphoma. JAK inhibitors, widely used for myeloid disorders, are now under investigation for treating patients with PTCL. Ruxolitinib appears to be the most promising, representing a potent JAK1 and JAK2 inhibitor that exerts its anti-JAK activity by competitive inhibition of the ATP-binding catalytic site of the kinase domain. Inhibition of the JAK-STAT pathway results in decreased STAT3/5, Akt, and ERK phosphorylation [79]. Durable responses were observed with ruxolitinib in a phase II study with a ~40% overall response rate in patients with activating JAK/STAT mutations or other evidence of JAK/STAT pathway activation [80]. 

### 8.7. Other Gene Alterations as Therapeutic Targets 

Twenty-four percent of ALK-negative ALCL shows aberrant levels of truncated Erb-B2 receptor tyrosine kinase 4 (ERBB4) transcripts, and ERB kinase inhibitors, such as cetuximab and gefitinib, are potential therapies. ERB kinase inhibitors inhibit the growth and survival of EGFR-expressing tumor cells [76]. 

Duvelisib is an oral inhibitor of phosphatidylinositol-3 protein kinase (PI3K) isoforms delta and gamma and is currently being tested in clinical trials for the treatment of T-Cell lymphoma. The PI3K pathway is critical for T-Cell differentiation, and derangements contribute to abnormal signaling and subsequent lymphomagenesis via lymphoma cell autonomy and tumor microenvironment modulation. Duvelisib may insert inhibition of the PI3K isoform activity by blocking mitogenic and survival signaling within the lymphoma cells or within the tumor microenvironment [60]. In one clinical trial, Horwitz et al. reported an overall response rate (ORR) of 50.0% in the PTCL population and 31.6% in the CTCL population. Responses were seen across the spectrum of PTCL subtypes with complete responses (CR) in patients with EATL, AITL, and PTCL-NOS, and partial responses (PR) in those with SPTCL, ALCL, AITL, and PTCL-NOS [81].

Finally, bortezomib, a protease inhibitor with NF-kB inhibitory activity, has shown some promise in relapsed or refractory aggressive adult T-Cell leukemia, which shows frequent gain-of-function alterations in the TCR/NF-kB signaling pathway due to an altered PLGC1 gene [82]. Fifteen patients were enrolled in the first stage of this study. One partial remission (PR) and five stable diseases (SD) were observed as the best overall responses, and the overall response rate (ORR) was 6.7% [82]. Potential genetic/molecular/protein targets for therapy are highlighted in Table 2.

## 9. Summary 

Peripheral T-Cell lymphomas represent a biologically and clinically heterogeneous disease where recent advances in gene expression profiling (GEP) and next-generation sequencing (NGS) have given insights into their complex molecular pathways.

With less than 1 case per 100,000 people in the United States, the rarity of PTCL provides diagnostic challenges for hematopathologists and clinical challenges for hematologists/oncologists [83]. Following expert hematopathology review, reclassification rates approached one third (AITL, PTCL-NOS) to one half (ALK-negative ALCL) for certain subtypes of PTCL [84]. To compound the diagnostic difficulty, the most common subtype of mature T-Cell lymphoma is PTCL-NOS, a diagnosis of exclusion sometimes referred to as a “wastebasket” category. A better understanding of genetic drivers in PTCL pathogenesis has enabled us to delineate different subtypes of PTCL and their prognostic implications.

Additionally, the body of information that has been amassed from GEP and NGS has identified potential targeted therapy options beyond the traditional CHOP/CHOP-like regimens, which include antibody-based therapy, histone deacetylase inhibitors (HDACi), chimeric antigen receptor T-Cells (CARTs), immune checkpoint inhibitors, and inhibitors targeting the TCR/co-stimulatory and JAK/STAT pathway. 

Given the aggressive nature of the disease and suboptimal success of standard treatment regimens, the identification of therapeutic targets beyond traditional treatment is a promising development. With so many potential targets being revealed, extensive clinical trials are required to examine the effectiveness of these novel therapies, alone and in combination with currently approved regimens. Hopefully, an individualized therapeutic approach will lead to durable clinical responses in the future.

## Figures and Tables

**Table 1:** Pertinent genetic and molecular aberrancies in different peripheral T-Cell lymphoma subtypes. The percentages in parentheses indicate the incidence of the aberrancy, where available. For abbreviations, please see the abbreviation list.

	Genetics and Molecular Aberrancies					
T-Cell Lymphoma Type	TCR, Co-Stimulatory, and Intracellular Signaling Pathway	Epigenetic Regulators	Tumor Suppressor Genes	Transcription Factors	Structural Alterations	Viral-Mediated
T-PLL			Missense or frameshift mutations in ATM (73%) and TP53 (71%)	Gain-of-function mutations in JAK3 (30–40%), STAT5B (21–36%), and JAK1 (8%)	inv(14) or t(14;14)(q11q32), t(X;14)(q28)(q11) (>90%), abnormal chromosomes 6,8 (amplification of MYC) (67%), and 17 (deletion of TP53), del(11q23)	
T-LGLL				Gain-of-function mutations in STAT3 (11–75%) and STAT5B (15–55%)		
ATLL	Activating mutations in PLCG1 (36%), VAV1 (18%), FYN (4%), CD28, and CTLA4-CD28 and ICOS-CD28 fusion genes (7%)		TP53 mutations (23%) CDKN2A deletions	Activating mutations in STAT3 (23%) and IRF4 (16%)		HTVL-1
HSTL		Nonsense and frameshift mutations in SETD2 (25%), INO80 (21%), TET3 (15%), SMARCA2 (10%)		Missense mutations STAT5B (31%) and STAT3 (9%)	isochromosome 7q (65%), trisomy 8 (50%)	
EATL				Mutations in JAK1, JAK3, and STAT3 (12–25%), less frequently STAT5B	9q34.3 gains (80%), 16q12.1 loss, 1q and 5q gains	
MEITL		Mutations in SETD2 (>90%)		Mutations in STAT5B (60%)	9q34.3 gains (75%), 16q12.1 loss (23%), gains of 8q (amplification of MYC) (50%)	
ENTKL		Mutations in KMT2D, KMT2C, ASXL3, ARID1A, and EP300 (4–18%)		Mutations in STAT3 and STAT5B (up to 35%)	del(6)(q21q25) (40%), loss of 6q16.1–27 (deletion of tumor suppressor genes PRDM1 or ATG5) (35%), 2q and 1p36.23–36.33 (40–60%)	EBV
STCLC		Mutations in KMT2D (17%)				EBV
MF/SS	Point mutations in PLCG1 (10%), CTLA4-CD28 and ICOS-CD28 fusion (3.6%)	Deleted DNMT3A (38%), ARID1A (58%), and ARID5B (29%)	Deleted TP53 (93%), CDKN2A (40%), RB1 (39%), and ATM (30%)	Copy number gains of STAT3 (60%), STAT5B (60%), and JAK2 (13%)		
AITL	Inactivating mutation in RHOA(G17V) (70%), FYN (4%) and VAV1 (5%) activating point mutations in CD28 (10%), CTLA4-CD28 (58%) and ICOS-CD28 fusion (5%)	Loss-of-function mutation in TET (80%), gain-of-function in IDH2 (20%), point mutation in DNMT3A(R882H) (30%)				
Follicular TCL		Loss-of-function mutation in TET2 (58%)			t(5;9)(q33q22) (ITK-SYK) (20%)	
ALK+ ALCL				STAT3 activation induced by t(2;v)(p23;v) fusion gene (13%)	t(2;v)(p23;v), most commonly t(2;5)(p23q35) (NPM-ALK) (85%)	
ALK- ALCL				Activating mutations in STAT3 and JAK1 (20%), NFKB2-ROS2 and NFKB2-TYK2 fusion genes contribute to STAT3 activation	t(6;7)(p25.3;q32.3)(DUSP22 -FRA7H) (30%), inv(3)(q26q28)(TP63-TBL1XR1) (8%), abnormal ERBB4 transcripts (24%)	
CD30+ PTCL				JAK2 rearrangements (most commonly with PCM1 as the fusion partner)		
Primary cutaneous ALCL					t(6;7)(p25.3;q32.3)(DUSP22-FRA7H) (33%), gains of 7q31, 17q, and 21, losses of 3p, 6q16–6q21, and 8p (52%)	
PTCL-GATA3			Loss or mutations of tumor suppressor genes in the CDKN2A/B (45%), TP53 (58%) and PI3K (35%) pathways	Gains and amplifications of STAT3 (35%)	Gains and amplifications of MYC (52%)	
PTCL-TBX21		Mutations in TET1, TET3, and DNMT3A (36%)		NF-kB activation		

**Table 2:** Potential therapy targets in peripheral T-Cell lymphomas. For abbreviations, please see the abbreviation list.

Genes	Potential Targets	Agents	T-Cell Lymphoma Type	References
NFAT pathway	Calcineurin	Cyclosporine A	AITL	[75]
*RHOA^G17V^, VAV1, FYN*	Multikinase inhibitors	Dasatinib	AITL, PTCL-NOS	[76,77,78]
PI3K pathway	PI3K isoforms gamma and delta	Duvelisib	Spectrum of PTCL	
*TET2, DNMT3A, IDH2*	HDACi	5-azacytidine, vorinostat, belinostat	AITL, ALCL, PTCL-NOS	[57,70,71,72,73,74]
*CTLA4-CD28* fusion, *ICOS-CD28* fusion	Anti-CTLA4 immunotherapy	Ipilmumab	AITL, PTCL-NOS	[69]
	PD-1, PD-L1	Nivolumab, prembrolizumab, atezolizumab, avelumab, durvalumab	AITL, PTCL with TFH phenotype	[68]
*JAK1, JAK2, STAT3, TYK2*	JAK/STAT inhibitors	Ruxolitinib	ALCL, ENKTL, cutaneous TCL	[79,80]
*ERBB4*	ERB kinase inhibitors	Cetuximab, gefitinib	ALCL	[76]
	NF-kB pathway	Bortezomib	ATLL	
	CD30	Brentuximab	ALCL and other CD30 expressing PTCLs	[58]
	CD52	Alemtuzumab (CAMPATH-1)	Any PTCL with CD52 expression	[59,60]
	CD25	Basiliximab, Camidanlumab	AITL, ATLL, ALCL, ENKTL	[62,63]
	CD38	Daratumumab	AITL, PTCL-NOS	[65]
	CCR4	Mogamulizumab	ATLL, ALK- ALCL, MF, AITL, PTCL-NOS	[66]
	CD7, CD4, CD5, CD30, TCR	CARs	Spectrum of PTCL	[60,67]

## Data Availability

Not applicable.

## Abbreviations

ADCC	antibody-dependent cellular cytotoxicity
ADCP	Antibody-dependent cellular phagocytosis
AITL	angioimmunoblastic T-Cell lymphoma
ALCL	anaplastic large cell lymphoma
ALK	anaplastic lymphoma kinase
ANKL	aggressive NK-cell leukemia
AP-1	activation protein 1
ARID	AT-rich interaction domain
ASXL3	ASXL transcriptional regulator 3
ATG5	autophagy-related gene 5
ATLL	adult T-Cell leukemia/lymphoma
ATM	ataxia telangiectasia mutated
ATP	adenosine triphosphate
BATF3	basic leucine zipper ATF-like transcription factor 3
BCL6	B-cell lymphoma 6
BV	brentuximab vedotin
CCR4	Chemokine receptor 4
CD	cluster of differentiation
CDKN	cyclin dependent kinase inhibitor
CHOP	cyclophosphamide, doxorubicin hydrochloride, vincristine sulfate, and prednisone
CNA	copy number alterations
CR	complete response
CTLA4	cytotoxic T-lymphocyte associated protein 4
D-2-HG	D-2 hydroxyglutarate
DNA	deoxyribonucleic acid
DNMT3A	DNA methyltransferase 3 alpha
DUSP22	dual-specificity phosphatase 22
EATL	enteropathy-associated T-Cell lymphoma
EBNA	Epstein-Barr nuclear antigen
EBV	Epstein-Barr virus
ENKTL	extranodal NK/T-Cell lymphoma
EP300	E1A binding protein P300
ERBB4	Erb-B2 receptor tyrosine kinase 4
FOXP3	forkhead box protein P3
FRA7H	fragile site, aphidicolin type, common, fra(7)(q32.3)
GATA3	GATA-binding protein 3
GPI	glycosylphosphatidylinositol
GTP	guanosine triphosphate
HCT	hematopoietic stem cell transplantation
HDACi	histone deacetylase inhibitors
HSTL	hepatosplenic T-Cell lymphoma
HTLV-1	human T-Cell leukemia virus type 1
ICI	immune checkpoint inhibitor
ICOS	inducible co-stimulator
IDH	isocitrate dehydrogenase
IFN	interferon
IL	interleukin
INO80	INO80 complex ATPase subunit
Inv(X)	inversion
ITK	inducible T-Cell kinase
ITLPD-GIT	indolent T-Cell lymphoproliferative disorder of the gastrointestinal tract
JAK	Janus kinase
KMT	lysine methyltransferase
KMT1	KMT1/Suv39 methyltransferase family
LMP	latent membrane proteins
LPD	lymphoproliferative disorder
MAPK	mitogen-activated protein kinase
MEITL	monomorphic epitheliotropic intestinal T-Cell lymphoma
MF	mycosis fungoides
MHC	major histocompatibility complex
MMAE	monomethyl auristatin E
MTCP1	mature T-Cell proliferation 1
MYC	MYC proto-oncogene
NFAT	nuclear factor of activated T-Cells
NF-kB	nuclear factor kappa-light-chain-enhancer of activated B cells
NGS	Next-generation sequencing
NK	natural killer
NOS	not otherwise specified
NPM	nucleophosmin
ORR	overall response rate
PCM1	pericentriolar material 1
PCR	polymerase chain reaction
PD-1	programmed death-receptor 1
PD-L1	programmed death-ligand 1
PFS	progression-free survival
PI3K	phosphatidylinositol-3 protein kinase
PIK3CD	phosphatidylinositol-4,5-bisphosphate 3-kinase catalytic subunit delta
PLC	phosphoinositide phospholipase C
PLGC1	phospholipase C gamma 1
PR	partial response or partial reponse
PRDM1	PR/SET Domain 1
PTCL	peripheral T-Cell lymphoma
PTEN	phosphatase and tensin homolog
PTPN	protein tyrosine phosphatase non-receptors
RB1	retinoblastoma 1
RHOA	ras homolog gene family, member A
RNA	ribonucleic acid
ROS1	ROS proto-oncogene 1
SD	stable disease
SETD2	Su(var), Enhancer of zeste, Trithorax(SET)-domain containing 2
SFKs	SRC family kinases
SH2	Src homology 2
SMARCA2	SWI/SNF-related, matrix-associated, actin-dependent regulator of chromatin subfamily A member 2
SS	Sezary syndrome
STAT	signal transducer and activator of transcription
STCLC	systemic EBV-positive T-Cell lymphoma of childhood
SYK	spleen tyrosine kinase
T(X;X)	translocation
T-bet	T-box expressed in T-Cells
TBL1XR1	TBL1X receptor 1
TBX21	T-box Transcription Factor 21
TCL1A	T-Cell leukemia/lymphoma protein 1A
TCL1B	T-Cell leukemia/lymphoma protein 1B
TCR	T-Cell receptor
TET	tet methylcytosine dioxygenase
TFH	T follicular helper
TH1	T-helper type 1
TH2	T-helper type 2
TMOD1	tropomodulin 1
TNFR	tumor necrosis factor receptor
TNFRSF8	TNF receptor superfamily member 8
TP	tumor protein
T-PLL	T-Cell prolymphocytic leukemia
TYK2	tyrosine kinase 2
WHO	World Health Organization
